# Atypical frontal lobe seizure as the first manifestation of gall-bladder cancer: a case report

**DOI:** 10.1186/s12883-019-1318-2

**Published:** 2019-05-10

**Authors:** Shweta Pandey, Ravindra Kumar Garg, Hardeep Singh Malhotra, Ravi Uniyal, Neeraj Kumar

**Affiliations:** Department of Neurology, King George Medical University, Lucknow, Uttar Pradesh 226003 India

**Keywords:** Ptosis, Seizure, Gall bladder cancer, Frontal lobe

## Abstract

**Background:**

Gall bladder cancer (GBC) is associated with abdominal pain, lump, nausea, vomiting, and jaundice due to either gall bladder mass or the involved adjacent peritoneal structures. Gall bladder cancer presenting as refractory epilepsy is rare. Here we report a young female GBC patient who presented with an atypical and refractory frontal lobe seizures as the first manifestation of gall bladder cancer.

**Case presentation:**

A 46 years young female presented first time to the hospital with uncontrolled seizures and headache in 5 months duration. Seizures were very atypical in semiology with ptosis and mydriasis to either side along with ipsilateral ocular deviation. The episodes were bilateral but right eyelid ptosis, mydriasis and right horizontal conjugate deviation were frequent. MRI brain showed encephalomalacia in the left frontal region on axial T2 and coronal T1 weighted images without any enhancement on gadolinium contrast. CECT abdomen revealed a heterogeneously enhancing gall bladder mass with the evidence of lung metastasis from chest CT scan. CSF for malignant cytology was negative. Seizures were refractory to the treatment.

**Conclusion:**

Though CNS involvement is uncommon but it can be the only presentation in gall bladder cancer.

**Electronic supplementary material:**

The online version of this article (10.1186/s12883-019-1318-2) contains supplementary material, which is available to authorized users.

## Background

The neurological manifestation in cancer appears because of the spread of the tumor to brain parenchyma or leptomeninges. CNS spread is uncommon in gall bladder cancer and only a few cases are reported till now [1, 2]. Stroke-like manifestation may be attributed to occlusion of pial vessels secondary to leptomeningeal involvement or a hypercoagulable state. Here, we report a patient with post infarction encephalomalacia in the left frontal area near frontal eye field leading to very atypical frontal lobe seizures and diagnosed later with gall bladder cancer.

## Case presentation

A 46 years old, previously healthy female presented with headache, fits and decreased vision for last 5 months. Headache was diffuse, non-throbbing, moderate intensity and occasionally associated with nausea and vomiting. The patient was suffering from headache every day without diurnal fluctuations. She was on daily analgesics for relieving the pain. A few days later she developed fits with eyes deviating to either side along with drooping of the eyelid and pupillary dilatation to the side where the eye would deviate. (Additional file 2: Movie S1) During the episodes, the patient responded partially and become very irritable afterward. The episode used to last for few minutes followed by irritable mood and crying for the next few hours (Additional file 3: Movie S2). The episodes were occurring daily. In most of the episodes forced eye deviation was to right with right eye ptosis. There was no history of fever, rashes and myoclonic jerks except significant loss of weight in the last 3 months.


**Additional file 2:** **Movie S1.** Video showing the ictus in which patient is having left eye-lid ptosis, mydriasis and conjugate eye deviation to left side. Patient is being asked to move her eyes to right but she is not following it. She is partially responding to commands with saying single word “YES” only. The episode is not associated with any other motor manifestation. (MP4 8970 kb)



**Additional file 3: Movie S2.** Video showing postictal state during which is patient is conscious, following commands and is having normal ocular movements without any ptosis. (MP4 6213 kb)


At admission, she was conscious, alert, higher mental functions were normal. Cranial nerves were normal. Bulk, tone, power and deep tendon reflexes were normal in both upper and lower limbs. There was no evidence of limb or gait ataxia. Bilateral plantar reflex was extensor.

**Fig. 1:**
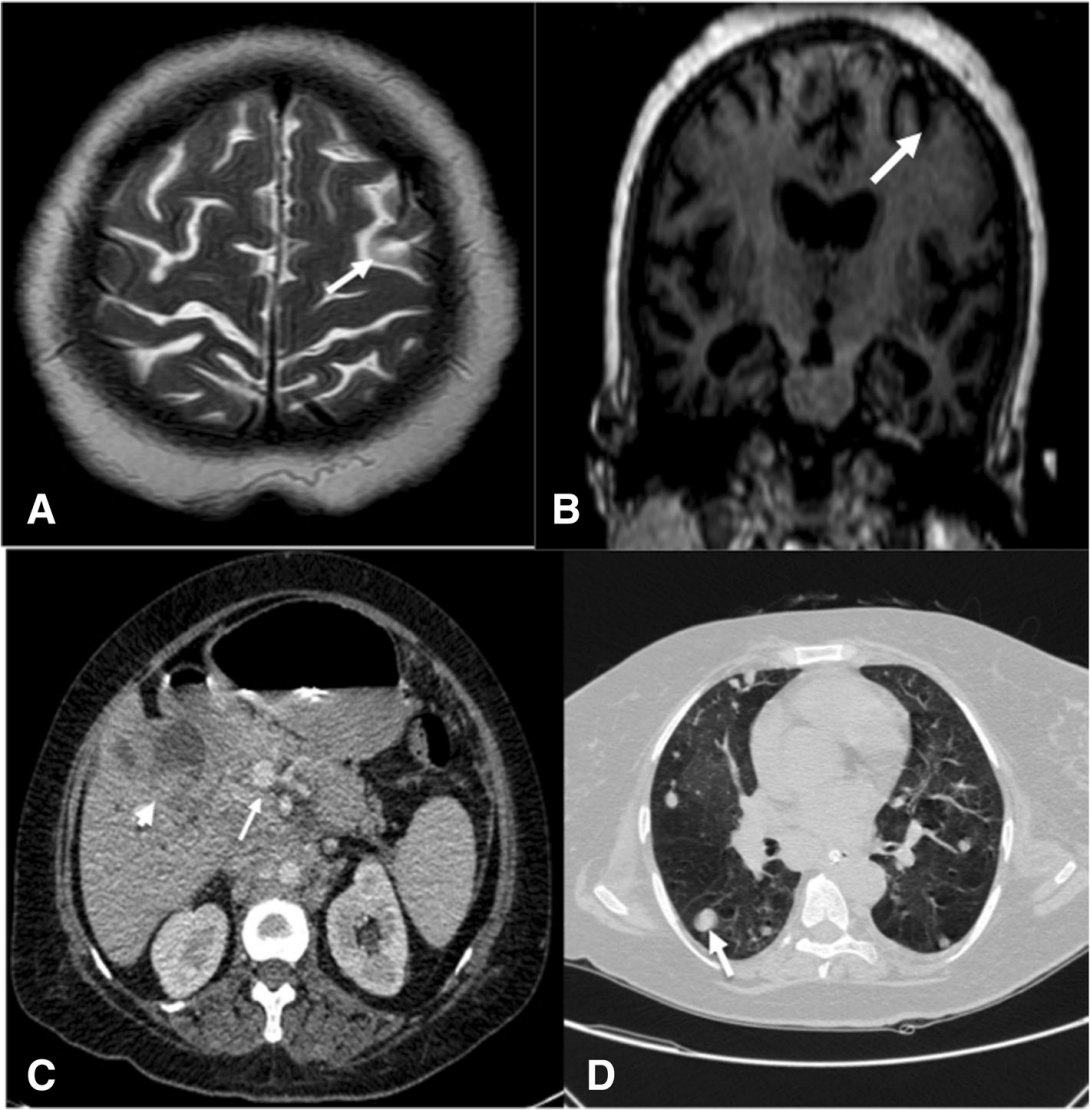
**a** MRI brain T2 weighted axial image showing encephalomalacia left frontal area with a corroborating signal intensity on T1 weighted coronal image (white arrow). **b**, **c** Contrast enhanced CT scan abdomen showing heterogeneously enhancing mass lesion in gall bladder (white arrow head) and enlarged periportal lymph node (white arrow). **d** CT thorax showing multiple metastatic lesion in lung parenchyma

Haemoglobin was 9.2 g% with microcytosis and anisocytosis. Total leucocyte count was 14,200/cu mm with neutrophil dominance. Liver functions were deranged with raised liver enzymes (SGOT-57 IU/L, SGPT-74 IU/L) and alkaline phosphatase (ALP-507 IU/L). Kidney functions, serum electrolytes and thyroid profile were normal. Her Serum ANA and ENA screen were negative. Chest X-ray showed haziness over the right lower zone. MRI brain axial T2 and coronal T1 weighted images revealed encephalomalacia left frontal area (Fig. 1). There was no enhancement on gadolinium contrast. Cerebrospinal fluid examination showed 20 cells all mononuclear, low sugar (55.6 mg% with corresponding blood sugar 145 mg %), and normal protein (41 mg %). CSF did not reveal any abnormal/immature cells. CSF PCR for Herpes Simplex Virus, varicella, enterovirus, Cytomegalovirus, and Japanese Encephalitis was negative. CSF gene expert for *Mycobacterium tuberculosis* was also negative.

The patient was started on intravenous antibiotics and loaded with phenytoin. As seizures were not controlled antiepileptics were gradually stepped up using levetiracetam, lacosamide, and clobazam in appropriate doses. Frequency of seizures reduced to 1–2/day. The patient was also given a course of intravenous methylprednisolone for 5 days.

As the patient had significant weight loss and abnormal chest X-ray, CT chest and abdomen was ordered to rule out malignancy. CT abdomen of the patient showed heterogeneously enhancing gall bladder mass and CT chest suggested multiple metastatic lesions in the lungs Fig. 1. Biopsy from cervical lymph node confirmed metastatic adenocarcinoma primary being gallbladder carcinoma (Additional file 1: Figure S1). Chemotherapy was planned but caregivers denied further treatment and took the patient home.

## Discussion

Seizure in systemic malignancy without CNS metastasis is reported uncommonly and focal seizures are even more unusual. Gallbladder cancer (GBC) is a very aggressive tumor with poor survival. It usually presents with local symptoms pertaining to the primary tumor and it spreads to liver, peritoneum, and lungs. Rarely, CNS involvement in GBC can be in the form of carcinomatous meningitis with features of raised intracranial pressure or space occupying parenchymal lesion with stroke-like features and/or focal seizures [3]. Seizure semiology in our patient with horizontal conjugate eye deviation to one side lateralizes the seizure to contralateral frontal eye field (FEF). Along with ocular deviation, our patient also had ipsilateral ptosis and mydriasis. The horizontal conjugate eye deviation, mydriasis and ptosis were changing sides in episodes but in the majority of the episodes, it was towards the right. Activation of area near FEF causes opening of eye-lids and lesions in these areas are reported to cause bilateral ptosis but more prominent on contralateral side [4]. Development of ptosis during an episode of seizure (irritative focus) is difficult to explain, however, it can be explained by the possible exhaustion of firing neurons and increased GABAergic activity due to repeated seizure episodes. We hypothesize that functional interruption of cortico-fugal fibers projecting from prefrontal region to midbrain may have led to both ptosis and mydriasis. It has earlier been described in some of the studies that unilateral pupillary dilatation can be a manifestation of frontal lobe seizures originating from middle frontal gyrus however lid ptosis was not reported in those patients [5].

Another possibility for this seizure semiology could be the spread of the seizure from frontal to the insular region resulting in ptosis and mydriasis as a part of autonomic manifestations of seizure.

The focal seizures in our patient probably resulted from the encephalomalacic zone in the left frontal area near frontal eye-field (Fig. 1). As the lesion was not enhancing on gadolinium contrast it could be a post infarct area. Development of ischemic infarct in a young patient with no vascular risk factors could be explained by the hypercoagulable state secondary to GBC. Although the hypercoagulable state in gall bladder cancer is rare and is commonly seen with cancers of pancreas, ovary, and lung. It is reported in adenocarcinoma of gall bladder cancer producing mucin [6]. The peculiarity of this case is that the patient with gall bladder cancer first time presented with refractory seizures with very atypical semiology and difficult to localize. EEG showed generalized slowing but epileptic focus could not be determined. An extensive and dedicated video-EEG along with Electrooculography (EOG) could have given a better answer which is a limitation in this case. Persistent cortical ptosis and seizures have been described in few clinical reports due to the hemispheric lesion but ptosis was not episodic and not described as part of seizure semiology like in our case [7]. Although we found post-infarct encephalomalacia in the left frontal area which could be a possible reason for the clinical presentation in our patient but the exact cause is still debatable.

## Conclusions

Neurological manifestations in a patient with gall bladder cancer although rare but can be the only presentation. Refractory de novo seizures in an adult do require workup for an underlying malignancy. Ptosis as a part of seizure semiology is not much described in the literature and further research is warranted in this area for clear anatomical localization.

## Additional files

Additional file 1: **Figure S1.** Lymph node biopsy: Hematoxylin & Eosin stain (400 X), showing tumor cells in cluster and sheets. Tumor cells are having pleomorphic nuclei, conspicuous nucleoli, and moderate amount of eosinophilic cytoplasm. Inset: Acini like arrangement of tumor cells (arrow). (PNG 2599 kb)

## Abbreviations

CECT: Abdomen Contrast Enhanced Computed Tomography of abdomen
FEF: Frontal Eye Field
GBC: Gall Bladder Cancer
