# Supplementary figures and images for: Atypical frontal lobe seizure as the first manifestation of gall-bladder cancer: a case report
Source: BMC Neurol. 2019 May 10;19:95. doi: 10.1186/s12883-019-1318-2 (PMC6511165; doi:10.1186/s12883-019-1318-2)

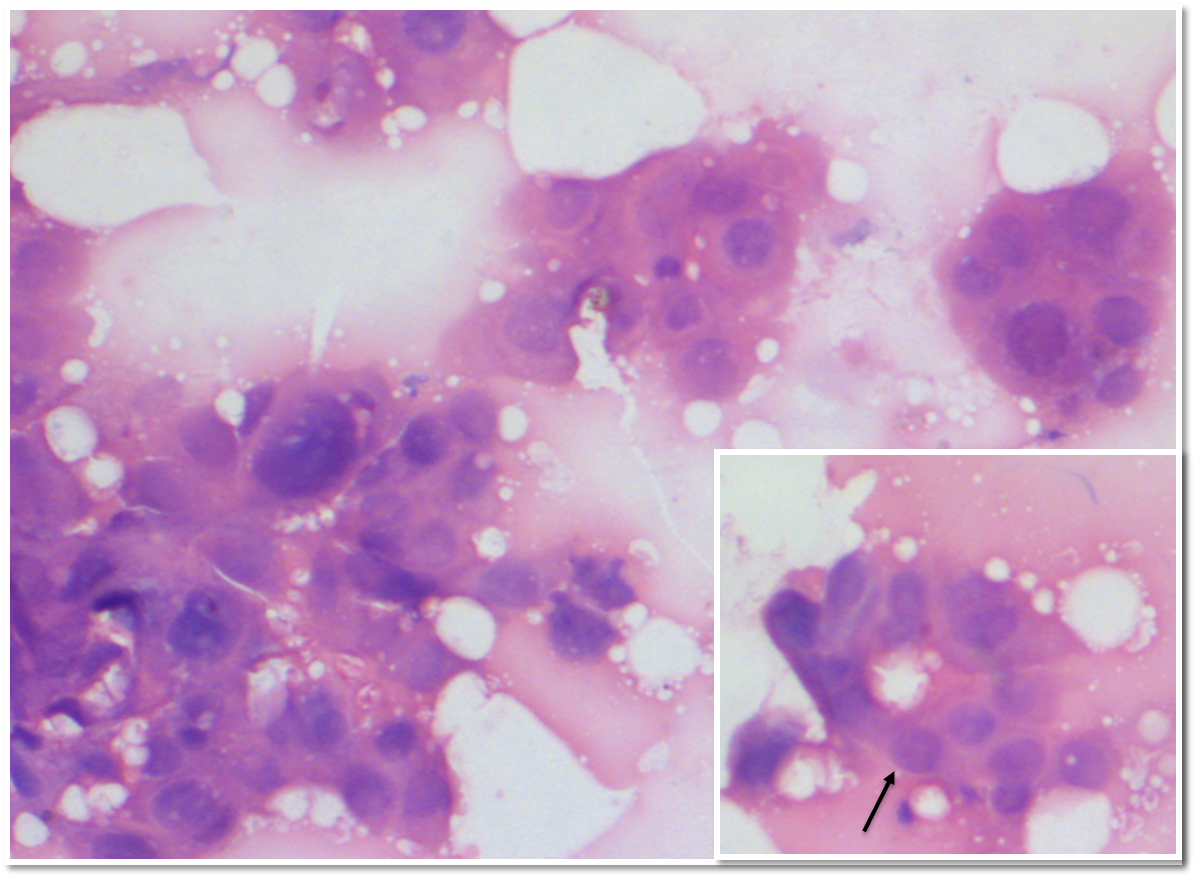

Supplement: Supplementary file 3 — Figure S1. Lymph node biopsy: Hematoxylin & Eosin stain (400 X), showing tumor cells in cluster and sheets. Tumor cells are having pleomorphic nuclei, conspicuous nucleoli, and moderate amount of eosinophilic cytoplasm. Inset: Acini like arrangement of tumor cells (arrow). (PNG 2599 kb) [file 12883_2019_1318_MOESM1_ESM.png]
